# Erratum: Correction of mortality outcome parameter

**DOI:** 10.29252/beat-070418

**Published:** 2019-10

**Authors:** R Ahl, Y Cao, H Geijer, K Taha, S Pourhossein-Sarmeh, P Talving, O Ljungqvist, S Mohseni

In the original article entitled “Prognostic Value of P-POSSUM and Osteopenia for Predicting Mortality After Emergency Laparotomy in Geriatric Patients” [1] which was published in July 2019 issue of the journal, there was an error in the specified mortality outcome in the manuscript. The published version describes 30-day mortality, however, the correct outcome measure should be 90-day mortality throughout the article. The article has been corrected accordingly online. 
